# Mental health and holistic care of migrant workers in Singapore during the COVID-19 pandemic

**DOI:** 10.7189/jogh.10.020332

**Published:** 2020-12

**Authors:** Lai Gwen Chan, Benjamin Kuan

**Affiliations:** 1 Department of Psychiatry, Tan Tock Seng Hospital, Singapore; 2 Medical Services, HealthServe, Singapore

Migrant worker populations in Singapore as well as other countries, despite advances in legislation and protections [1], continue to face longstanding issues of barriers to equal access to health care [2], information and resources targeted at the host country’s local population, and even exclusion from national crisis response plans particularly pandemic preparedness plans [3]. Non-governmental organizations (NGOs) have a vital role in filling this gap by providing accessible services (eg, welfare, health care, crisis), liaison between employers and financing agents, and advocacy by representation of their needs and concerns in multi-agency collaborations.

Specifically, mental health care for this population has been lacking because language and cultural barriers make it challenging for general health care services to incorporate this aspect. High levels of stigma towards mental illness in the home countries of migrant workers also add to the burden of unmet needs for focused mental health support. The scarcity of such services even among the NGO sector became amplified during the COVID-19 pandemic in Singapore when mass quarantine and isolation of migrant worker accommodations severely reduced NGOs’ access to them.

HealthServe [4] was one such NGO which, in the 14 years since it was founded, has established strong networks of friendship and goodwill between ministerial agencies, employers, migrant worker dormitory operators and migrant workers. HealthServe operates low-cost primary care clinics including case work support to workers who had sustained workplace injuries, and commenced a mental health and counselling department, the first among the other migrant worker NGOs, in 2019.

## MEASURES TAKEN

When news broke in early April 2020 about the gazetting of 2 large migrant worker dormitories as isolation areas because of clusters of confirmed COVID-19 cases there [5], HealthServe anticipated the unprecedented magnitude of mental health and psychosocial care needs and stepped forwards with a manual of recommendations on how to engage the migrant worker population as well as how to address the mental health and psychosocial care needs (an adaptation and application of the Interim Briefing Note Addressing Mental Health and Psychosocial Aspects of COVID-19 Outbreak developed by the IASC’s Reference Group on Mental Health and Psychosocial Support in Emergency Settings [6]). (**Table 1**). This was circulated to governmental and community teams who were involved in migrant worker outreach. It describes a tiered model of care where the recommended interventions get increasingly specific and specialized as one moves up the tiers. For example, Tier 1 recommends basic communication and connection strategies to meet basic needs for safety, and the highest tier describes specialized mental health services delivered by mental health professionals. This manual continues to be an evolving document as more is learnt and as policies change in real-time.

**Table 1:** Recommended tiered model of mental health and psychosocial support

Tier of Intervention Pyramid	Interventions
**Tier 1**	• enable autonomy over simple daily routines that are culturally and religiously appropriate:
	- provide resources/equipment needed to maintain dignity in lifestyle eg, housekeeping necessities, toiletries, food for cooking comfort foods (within appropriate social distancing measures & contexts)
Social considerations in basic services and security	• provide freedom, space, resources for religious rituals
	• ensure basic, positive daily communications through any broadcasting system in appropriate languages
Basic themes in establishing sense of safety:	• give adequate advanced notice and information about any upcoming transitions, eg:
	- transfers from dormitory to hospital or isolation facility or discharge back
	- explain their medical condition, reason for transition, what to expect after transition etc
	• repeat and reinforce information post-transition similarly
• sensual comforts (eg, preferred foods)	• have staff trained in ***Psychological First Aid*** to Look, Listen and Link
	• provide a means for feedback and concerns to be raised and addressed eg,
	- message boards,
	- WhatsApp chat groups,
	- daily face-to-face check-ins, and
	- ensure timely updates on the concerns raised
• habitual comforts (eg, familiar routines)	• provide information and access to COVID19 specific information, eg, https://covid19.HealthServe.org.sg/
	• provide information and access to self-help resources, eg, HealthServe hotline
• hearing and feeling heard	• written information in the form of care cards or health booklets can be helpful but **audio-visual forms are preferred** because verbal comprehension abilities are generally stronger than reading comprehension
•	• ensure access to chronic disease management
**Tier 2**	• HealthServe website to be regularly refreshed with mental wellness content
	• Encourage self-enablement and empowerment
	• Provide resources for suggested appropriate pleasurable activities, eg,
	- games (playing cards, carrom, chess, etc)
	- craft work, art/drawing
	- outdoor movie screening
	- etc
Strengthening personal resilience and community and family supports – activating social networks, supportive spaces	• Plan special activities, foods, occasions to be looked forward to
	• Enable celebration of significant cultural/religious festivals in appropriate ways
	• Encourage helping and looking out for each other who are in the same cohorted space, and have autonomy over their space (eg, ‘Cleanest Room / Corridor / Block’ competitions)
Basic themes:	• Encourage ground-up initiatives and ideas on activity scheduling and system improvements
• Connection	
• communication	• Announce and celebrate achievements
• contribution	• Harness technology to enable new ways of expression, communications and communal activities
• meaning	
**Tier 3**	**Hospitalized or quarantined workers**
	• Prepare a pool of available translators and have a low threshold for calling on them for help
	• develop communications scripts for use between patient/Healthcare Worker and Healthcare Worker/patient’s Next of Kin
	• include aspects from Tier 1 and Tier 2 in admissions orientation (such as sources of information and self-help)
Non-specialised supports - basic emotional and practical support by community workers	• posters and informational materials on **HealthServe’s hotline** to be displayed prominently for awareness and self-help
	• referral pathways for onsite teams to refer cases to HealthServe Tele-Befriender service or onsite teams to allow access to HealthServe to provide outreach
	- **all attending teams (medical and non-medical) are advised to proactively detect signs of emotional distress**
	**Dormitory workers**
HealthServe “Tele-Befriender” service in the form of 1:1 or group sessions, fronted by volunteers and supervised by mental health experts	• Posters and informational materials on **HealthServe’s hotline** to be displayed prominently at medical post
	• referral pathways for onsite teams to refer cases to HealthServe Tele-Befriender service or onsite teams to allow access to HealthServe to provide outreach
	- **all attending teams (medical and non-medical) are advised to proactively detect signs of emotional distress**
	• employers and dormitory managers can also refer to HealthServe Tele-Befriender service by sending a text message to the **HealthServe hotline**
**Tier 4**	• Cross-referral across health care institutions to accord appropriate care for individuals requiring more intense intervention
Specialised services – mental health care by mental health specialists	• Early involvement of skilled resource from tertiary hospitals and national institutes for just-in-time care
	• **Proposed workflows for referral and escalation (to be developed according to jurisdiction)**

**Figure:**
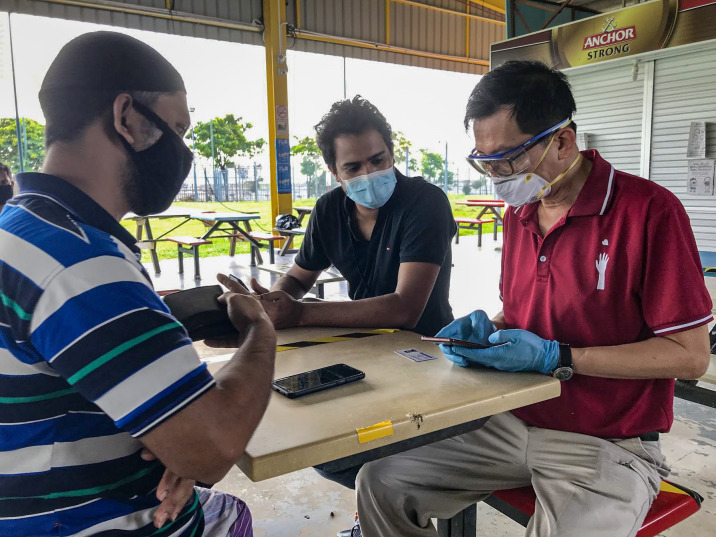
Photo: HealthServe staff providing psychosocial assistance to workers in a gazetted dormitory and enabling connectivity via smartphone (from the authors’ collection, used with permission).

## DISCUSSION

Additional strategies were adopted by HealthServe and these are the ones with the greatest impact:

1. Proactively approaching the Inter-Agency Task Force [7] that had been set up to provide support to dormitory operators and the migrant workers living there in order to be a representative voice for migrant workers as well as offer recommendations on how best to engage this population. This was made possible through pre-existing networks with ministerial agencies and a high level of trust between parties. This collaboration continues to be active and deepen and ensured that mental health care continues to be a priority.

2. Partnered medical teams providing on-site medical care to dormitories to conduct small group engagement with migrant workers for needs assessment. Concerns and feedback were systematically gathered and resulted in rapid improvements to care and services provided on the ground as well as future planning. These concerns included issues related to food provision, fears of COVID-19 transmission, lack of timely information, fears of deportation etc.

3. Massive call for volunteers with and without mental health expertise to enlarge the pool of native speakers, and training all in Psychological First Aid as well as basic knowledge about possible emotional responses and issues faced by migrant workers.

4. Point (3) was concurrent with the setting up of a crisis hotline and a web-based registration page where migrant workers could register to receive support through a Tele-Befriending virtual clinic fronted by trained volunteers and supervised by a volunteer psychiatrist. This virtual clinic has already reached out to hundreds of migrant workers.

5. HealthServe’s Communications Team also set up a specific COVID-19 information webpage [8] accessible in different languages of the migrant workers, with information regarding the pandemic, social support that is available, as well as mental wellness information. A separate team of volunteers also prepared a pipeline of content to regularly refresh the webpage. From experience, migrant workers tended to use social media platforms more frequently, hence HealthServe also ensured its social media pages [9] were regularly refreshed with content and the relevant links to further online information. HealthServe also engaged the partnership of the country’s major telecommunications providers to send out SMS (short messaging system) blasts pointing the migrant worker population to these online resources and worked with social media companies to target relevant information specific to migrant workers. One key learning was that this population tended to consume information via video and audio rather than text and HealthServe hence worked closely with media houses to produce content in these formats. Videos featuring celebrities from the workers’ home countries were particularly well-received.

6. Outreach to and partnerships were made with the National Centre for Infectious Diseases (NCID, where the bulk of COVID-19 cases were given acute care) and the community isolation facilities (where COVID-19 patients were decanted from acute hospitals) so as to provide support on how to apply the manual’s recommendations in the specific settings. For example, creating videos to be broadcast in hospital rooms on what to expect in their journey as a patient traversing through the health care system, creating simple and pictorial health booklets in native languages to empower migrant workers to take charge of their own health and mental well-being, setting up of remittance booths/kiosks for online remittance, supporting Muslim migrant workers who wanted to observe Ramadhan (setting up prayer corners, providing prayer mats, specific foods and timings to break fast), and even encouraging ground-up initiatives such as providing haircuts. There was much positive qualitative feedback about these.

7. Psychiatric departments of different hospitals and the country’s tertiary psychiatric hospital also reached out to establish partnerships, referral pathways and escalation protocols for migrant workers identified to require more specialised mental health assessment and interventions. These have proven to be useful especially as cases of major mental illness and serious self-harm began to emerge and the numbers requiring psychiatric intervention are rising.

8. HealthServe also actively “walked the ground” for outreach to migrant workers who were not living in established purpose-built dormitories. Such workers were usually living in factory-converted dormitories or private rental apartments, and a smaller group were those given a special permit to remain in Singapore while awaiting workmen’s injury compensation or salary disputes to be resolved. This had the impact of reaching even the most marginalized migrant workers who would otherwise have fallen through the cracks in the system. “Walking the ground” also provided opportunities to engage with the dormitory teams and managing agents of care facilities, who did not have prior experience with engaging migrant workers, to build trust and establish the informal networks that were essential for access and timely responses.

9. Chronic disease management began to emerge as an important health care need for discharged migrant workers who had been diagnosed whilst being treated for COVID-19. HealthServe agreed to receive these referrals to its primary care clinics and worked with the different health care providers on referrals and continuity of care. This aspect was also identified as a health care need that will continue to exist beyond the COVID-19 pandemic and will require further conversation with stakeholders and policy changes regarding their health care financing.

10. Finally, continued mental health commitment by the HealthServe board beyond COVID-19 was important for both partners and management. The Board determined that mental health services would be needed for the longer term to not only address the longer term sequelae of COVID-19 but also to respond to the heightened awareness of mental health and psychological well-being of the migrant workers and committed further resources to sustain the services established during this time and to expand them.

It is now almost three months since the spike in COVID-19 cases among migrant workers, and mental health and suicide prevention have now become one of the key priorities on the Task Force’s agenda. This experience has demonstrated how a medical NGO can engage in a functional, effective, and evolving collaboration with both governmental and non-governmental stakeholders as recommended by the World Health Organization in its Interim Guidance [10], so as to ensure that the voice of the migrant worker population is represented and balanced policies regarding their management are made. It is neither too early nor too late to address mental health and psychosocial concerns of the migrant worker population. Nevertheless, it is hoped that this capacity would be built in sooner rather than later in future pandemics and that this paper would serve as a crucial guidance for other jurisdictions.
